# An Outbreak of Acute Hepatitis in a Medical Facility of Bangladesh

**DOI:** 10.5005/jp-journals-10018-1102

**Published:** 2014-01-22

**Authors:** Mamun Al Mahtab, Sheikh Mohammad Fazle Akbar, Dulal chandra Podder, Paban Kumar Saha, Munira Jahan, Lovely Begum, Tanjina Afrose, Farzana chowdhury, Salimur Rahman

**Affiliations:** 1 Department of Hepatology, Bangabandhu Sheikh Mujib Medical University, Dhaka, Bangladesh; 2 Department of Medical Sciences, Toshiba General Hospital, Tokyo, Japan; 3 Department of Medicine, Kumudini Hospital, Tangail, Bangladesh; 4 Department of Medicine, Kumudini Women’s Medical College and Hospital, Tangail, Bangladesh; 5 Department of Virology, Bangabandhu Sheikh Mujib Medical University, Dhaka, Bangladesh; 6 Research Division, Catalysis South Asia Operations, Dhaka, Bangladesh; 7 Department of Medicine, Kidney Foundation Hospital and Research Institute, Dhaka, Bangladesh; 8 Department of Anesthesiology, Ad-din Medical College and Hospital, Dhaka, Bangladesh; 9 Department of Hepatology, Bangabandhu Sheikh Mujib Medical University, Dhaka, Bangladesh

**Keywords:** Acute icteric hepatitis, Bangladesh, Medical facility, HEV, Viral marker.

## Abstract

A total of 45 patients with acute hepatitis were detected in a medical facility of Bangladesh over a period of 6 months. All of them were physicians, nurses, students or employees of the hospital. About 50% of these patients suffered from acute hepatitis within a period of 2 months. All of them had clinical and biochemical evidences of acute hepatitis. All of them shared common working places as well as common dining and cooking facilities. Although the disease was supposed to be caused by hepatitis viruses, none of them were expressing IgM type antibody to hepatitis B core antigen (IgM anti-HBc) or hepatitis C virus (IgM anti-HCV). IgM type antibody to hepatitis A virus (IgM HAV) was detected in one patient and IgM type antibody to hepatitis E virus (anti-HEV IgM) were found in 14 patients. In conclusion, diagnosis of etiological agent of viral acute hepatitis constitutes a formidable challenge to the existing health care delivery system in developing countries as available serological and routine screening fails to find the proper etiological agent.

**How to cite this article:** Mahtab MA, Akbar SMF, Podder DC, Saha PK, Jahan M, Begum L, Afrose T, Chowdhury F, Rahman S. An Outbreak of Acute Hepatitis in a Medical Facility of Bangladesh. Euroasian J Hepato-Gastroenterol 2014;4(1):66-67.

## INTRODUCTION

Acute icteric hepatitis due to hepatitis viruses is common in most developing countries of Asia and Africa. Most of these countries are yet to ensure supply of safe drinking water and proper disposal of sewerage. Based on these facts, it is assumed that acute icteric hepatitis is mainly caused by HAV or/and HEV in these countries, although evidences are lacking to confirm these assumption. Most of the patients with acute hepatitis seldom appear to physicians or attend medical facilities. Only patients with severe acute hepatitis attend physicians as this constitute a medical emergency.

We experienced an outbreak of acute icteric hepatitis at Kumudini Hospital, Tangail, Bangladesh, in 2011. The outbreak started in February 2011 and continued until August 2011. A total of 45 patients exhibited the features of acute hepatitis. All ofthem were concentrated in Kumudini Hospital, either as doctors (N = 18), or nurses (N = 4) or students (N = 11) or hospital workers (N = 12). The age of patients varied from 19 to 38 years. Thirteen patients had history of jaundice, although the etiological factors remain unknown. Most of the patients shared common drinking water, common cooking and common dining facilities. In 15 cases, at least two persons living together in a single room presented with acute hepatitis. The patients presented with typical symptoms of acute hepatitis, such as nausea, vomiting, abdominal pain, fever, itching, constipation and dyspepsia. Also, weakness, anorexia, diarrhea, malaise, vertigo and yellow urine were reported by some patients as presenting symptoms. Also, biochemical investigations of serum bilirubin, serum alanine aminotransferase, aspartate aminotransferase supported the diagnosis of acute hepatitis. The cause of acute hepatitis was not HBV or HCV because all of them were negative for IgM anti-HBc and IgM anti-HCV. IgM anti-HAV was detected in only one patient and IgM anti-HEV was detected in 15 patients. Thus, we assumed that the outbreak of acute hepatitis in Kumudini Hospital may be attributable to HEV.

The present study was conducted to have insights about etiology of acute icteric hepatitis that broke out within a hospital premise at Bangladesh. There was no other outbreak of acute icteric hepatitis at Tangail district at that time and this was confirmed from district health authorities as well as from newspaper sources. Thus, it is predictable that contaminated foods or drinks were responsible for such an outbreak. In fact, 14 of 45 patients exhibited IgM anti-HEV indicating that the outbreak was due to HEV. However, IgM anti-HEV could not be detected in 30 patients of this cohort. It was interesting to note find that two persons from the same room showed acute hepatitis within a span of 1 week. However, IgM anti-HEV was detected from sera of one person and could not be detected from the sera of other.

This study has raised several questions about diagnosis of acute hepatitis and acute hepatitis E, especially about the etiological factors of acute hepatitis. From the circumstantial evidences, it seems that the outbreak was mainly due to HEV. However, the proper etiological agent could not be detected in more than 60% patients. However, this is not the first study that showed some data of this nature. Although presumed to be acute hepatitis due to hepatotrophic viruses, Chandra et al have shown that no viral marker could be detected in 32% patients with acute viral hepatitis in their cohort.^1^ An Italian study also showed that acute hepatitis E can be reliably detected in only 44.2% patients.^2^ On the other hand, Huang et al have commented that it is not practicable to diagnose the true occurrence of acute hepatitis E and they also suggested that assessment of all possible markers would be required to establish a diagnosis of acute hepatitis E.^3^

In fact, we also had major limitation study designing. The first, sera were collected from these patients only once and that may not be proper timing for anti-HEV IgM positivity or HEV RNA expression. Thus, multiple sera samples should be collected for proper assessment of etiology of sporadic and endemic acute hepatitis. The next, there has been significant controversies about implication of IgM anti-HEV and acute hepatitis E. Recently, IgA type antibody to HEV appears to be better marker of acute HEV infection than IgM anti-HEV. A study from Japan has found that a patient of acute hepatitis E never expressed IgM anti-HEV but expressed IgA anti-HEV.^4^ Thus, improvement of clinical design and assessment of IgA anti-HEV would improve diagnosis modality of HEV infection in acute hepatitis patients. Also, attention should be given to estimation of HEV antigen for diagnosis of acute hepatitis E because they are expressed prior to expression of antibody to HEV.^5^
